# Robotic Approach to Paediatric Gastrointestinal Diseases: A Systematic Review

**DOI:** 10.3390/children11030273

**Published:** 2024-02-22

**Authors:** Rauand Duhoky, Harry Claxton, Guglielmo Niccolò Piozzi, Jim S. Khan

**Affiliations:** 1 Department of Colorectal Surgery, Portsmouth Hospitals University NHS Trust, Portsmouth PO6 3LY, UK; rauand.duhoky@porthosp.nhs.uk (R.D.); guglielmo.piozzi@porthosp.nhs.uk (G.N.P.); 2 School of Computing, Faculty of Technology, University of Portsmouth, Portsmouth PO1 2UP, UK; 3 Department of Paediatric Surgery, University Hospital Southampton NHS Foundation Trust, Southampton SO16 6YD, UK; 4 Faculty of Science and Health, University of Portsmouth, Portsmouth PO1 2UP, UK

**Keywords:** gastrointestinal disease, paediatric surgery, robotic surgery, children, colorectal, congenital

## Abstract

Introduction: The use of minimally invasive surgery (MIS) for paediatric surgery has been on the rise since the early 2000s and is complicated by factors unique to paediatric surgery. The rise of robotic surgery has presented an opportunity in MIS for children, and recent developments in the reductions in port sizes and single-port surgery offer promising prospects. This study aimed to present a systematic overview and analysis of the existing literature around the use of robotic platforms in the treatment of paediatric gastrointestinal diseases. Materials and Methods: In accordance with the PRISMA Statement, a systematic review on paediatric robotic gastrointestinal surgery was conducted on Pubmed, Cochrane, and Scopus. A critical appraisal of the study was performed using the Newcastle Ottawa Scale. Results: Fifteen studies were included, of which seven were on Hirschsprung’s disease and eight on other indications. Included studies were heterogeneous in their populations, age, and sex, but all reported low incidences of intraoperative complications and conversions in their robotic cohorts. Only one study reported on a comparator cohort, with a longer operative time in the robotic cohort (180 vs. 152 and 156 min, *p* < 0.001), but no significant differences in blood loss, length of stay, intraoperative complications, postoperative complications, or conversion. Conclusions: Robotic surgery may play a role in the treatment of paediatric gastrointestinal diseases. There is limited data available on modern robotic platforms and almost no comparative data between any robotic platforms and conventional minimally invasive approaches. Further technological developments and research are needed to enhance our understanding of the potential that robotics may hold for the field of paediatric surgery.

## 1. Introduction

The use of minimally invasive surgery (MIS) for paediatric surgery has been on the rise since the early 2000s, with a substantial body of supporting literature across several specialties [1,2,3,4,5]. MIS in children is complicated by unique challenges inherent to the paediatric setting, such as instrument/port size, limited working space intra- and extracorporeally, and fragility of tissues. The rise of robotic surgery in the early 2000s has presented a potential development in the MIS treatment of children, and recent developments in the reductions in port sizes and the progression in single-port surgery can be promising. The technical advantages of the robotic approach include an increased range of instrument movement due to articulated instruments, a fixed surgeon-controlled 3D camera for a stable operative view, an ability to filter out tremors, and the optimisation of plane dissection through a surgeon-controlled third arm [6].

The first publication on robotic paediatric surgery was on Nissen fundoplications on a 10- and a 12-year old patient in Germany in 2001 [7]. As robotic surgery has expanded worldwide and through different surgical subspecialties, so it has in paediatric surgery, with over 2000 robotic paediatric surgeries performed a decade after the first publication [8]. These surgeries include gastrointestinal, genitourinary, and thoracic procedures, and the number of robotic paediatric surgical cases have only continued to increase, with reports of a 7.5% increase every year [9]. However, the use of robotic surgery in children has not kept pace with its application in adults. The rarity of paediatric diseases and the limited access to the platform, often used by other surgical specialties, have resulted in a scarcity of high-quality data and publications [8]. Retrospective reviews show upper gastrointestinal surgery to be the most common procedure performed robotically on children, with cholecystectomies and Nissen fundoplications at the top of the list [9].

Despite the potential benefits of using robotics on children, the costs of the platform remain one of the biggest concerns, with some publications showing higher costs compared to open and laparoscopic approaches [10]. Similarly to the use of robotics in adult surgery, however, supporters of the technique argue that some of the costs of robotics could be reduced through more efficient, coordinated programs, and the appropriate training of both surgeons and theatre staff [11]. Lastly, there have been some concerns about the instrument sizes used with robotics, as most paediatric surgeons prefer using 3 mm instruments, but robotic instruments are currently available only in an 8 mm format, though more and more platforms are reaching the market every day, and the technology suggests a promising trajectory.

To further implement robotics in paediatric gastrointestinal surgery it is essential to summarise the current state-of-the-art robotics and draw conclusions on their roles. This study aimed to report the perioperative and functional outcomes of paediatric gastrointestinal diseases treated with a robotic approach through a systematic review of the literature.

## 2. Materials and Methods

The systematic review was conducted according to the 2020 guidelines of the PRISMA Statement (Preferred Reporting Items for Systematic Reviews and Meta-Analyses) [12]. All the PRISMA steps were performed independently by two authors (RD and HC). Any discrepancies were resolved between the reviewers, and the final decisions on eligibility were solved through consensus.

### 2.1. Search Strategy and Eligibility Criteria

#### 2.1.1. Identification

This study was registered on PROSPERO (#CRD42023472290), and a systematic literature search was performed using the PubMed, Cochrane, and Scopus databases. The following search terms were used: (pediatric OR paediatric OR children) AND (surgery OR surgical) AND (gastrointestinal OR gastro-intestinal OR GE OR GI OR bowel OR colorectal OR digest*) AND (robot*). The results were restricted to publications after 2000, as the development of robotic platforms did not reach maturity until the early 2000s, and any publications from before then are not reflective of current systems. The search results were pooled, and duplicates were removed. Reference lists from the retrieved manuscripts were reviewed to identify additional relevant articles.

#### 2.1.2. Screening and Eligibility

The studies were screened in two phases: title and abstract, and full-text screening. Non-retrievable studies were excluded. The inclusion criteria were as follows: (1) children ≤ 12 years old; (2) gastrointestinal diseases or disorders; and (3) robotic-assisted surgery. The exclusion criteria were as follows: (1) children > 12 years old and adults; (2) non-surgical treatment; (3) incorrect study design (conference abstracts, unpublished manuscripts, animal studies, editorials, video vignettes, and comments); and (4) full text written in any language other than English. In the case of more than one study published by the same authors with overlapping data or periods, the study with the more adequate design was considered for the review.

### 2.2. Data Extraction

The following study and patient data were collected: first author, publication year, country where the study was performed, study design, sample size, time period of data collection, age, type of disease, robotic platform, and generation of robot.

The following study outcomes were collected: surgical technique, procedure, age, operative time (OT), intraoperative complications (IOCs), length of stay (LOS), postoperative complications (POCs), conversion (defined as any unplanned extension of the extraction site during surgery), re-admissions, re-interventions, and follow-up. 

Data extraction was performed independently by two authors (RD and HC). Any discrepancies and the final decision on the data were solved through consensus. Missing data were reported as NA (Not Available). The Rayyan web and mobile app was used for the screening process [13].

### 2.3. Methodological Quality Appraisal

A critical appraisal of the study quality (risk of bias assessment) was independently performed by two authors (RD and HC) according to the Newcastle Ottawa Quality Assessment Scale (NOS) for Cohort and Case Control Studies [14]. No predetermined criteria for exclusion were defined. Any discrepancies were solved through consensus to reach a final decision.

### 2.4. Statistics

Categorical data were expressed as absolute values and/or pooled percentages. Continuous data were expressed as the absolute mean with standard deviation for normally distributed data or median values with interquartile ranges for non-normally distributed data. Bivariate categorical data were analysed using a chi-square test or Fisher’s exact test. All other categorical data were analysed using Fisher’s exact test. Post-hoc analyses were performed using either a Bonferroni correction test or nominal symmetry test to determine which sub-analyses were significant. Numerical data were analysed using either an unpaired *T*-test or the Mann–Whitney U test, depending on the distribution of data. Kaplan–Meier curves or cox regression analysis were used for calculating time-to-event data. Statistical analyses were conducted using IBM SPSS Statistics for Windows, version 28 (IBM Corp., Armonk, NY, USA) and R version 4.1.2 (R Foundation for Statistical Computing, Vienna, Austria).

## 3. Results

### 3.1. Study Characteristics

**Figure 1:**
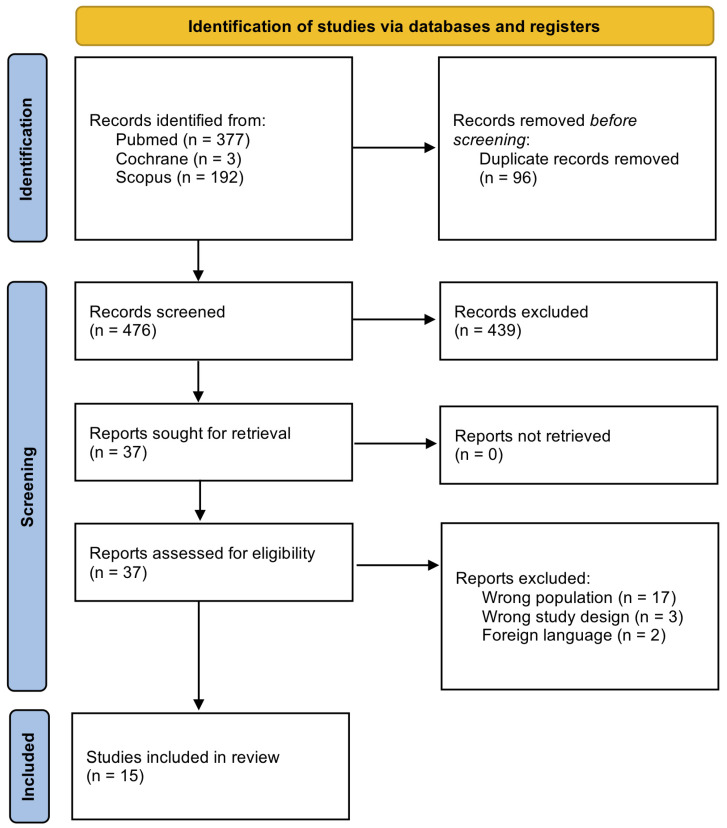
PRISMA study flow diagram.

The systematic search identified 476 studies. After removing duplicates and title and abstract screening, 35 studies were assessed for eligibility through full-text evaluations. In the end, fifteen studies were considered eligible and included in the review (Figure 1) [15,16,17,18,19,20,21,22,23,24,25,26,27,28,29].

### 3.2. Gastrointestinal Diseases

Of the fifteen included studies, seven publications were on the primary treatment of Hirschsprung’s disease, one on redos for Hirschsprung, two on anorectal malformations (ARMs), and the remaining five covered a multitude of indications (Table 1). One paper provided comparative analyses, the remaining fourteen provided only descriptive data of their populations. The studies included populations from five countries, with China and Italy having four publications each; the United States and Saudi Arabia, three publications each; and Vietnam with one publication. There were three prospective cohorts (one prospective multi-centre and two prospective single-centre), and the remaining twelve were retrospective cohorts. The total accumulative sample size across all fifteen studies was 470 patients. 

### 3.3. Hirschsprung’s Disease

The seven publications on Hirschsprung’s disease are shown in Table 2. Except for Hebra et al. [19], who used the Swenson technique, all publications utilised the Soave technique for their surgical approach. The average age at operation varied between 3.7 and 24.5 months, and reported patients were predominantly male (50.0–80.0%). The total OT and LOS varied significantly between the studies, ranging from 93.2 to 372 min and from 3 to 8.8 days, respectively. There were no IOCs in most included studies, except for Hebra et al.’s with one (8.3%) and Prato et al.’s (2019) with four (44.4%) [19,26]. No conversions were reported in any of the robotic cohorts, but the laparoscopic and transumbilical laparoscopy single-site surgery (TU-LESS) cohort in Li et al.’s study each had one conversion [17]. POCs ranged from 16.0% to 33.3%, and readmission and reintervention rates were low in all studies (0% to 9.4% and 0% to 6.7%, respectively). Faecal incontinence ranged from 0% to 22.2%, with 7/9 studies reporting less than 7% incontinence within their follow-up. Follow-ups ranged from 4.5 to 79 months across the included studies [15,17,18,19,22,24,26].

### 3.4. Other Diseases

The remaining publications varied significantly in their surgical indications and are shown in Table 3. Indications included achalasia, ARMs, mesenteric cysts, Nissen fundoplications, cholecystectomies, redos for Hirschsprung’s disease, and restorative proctocolectomies. Ages ranged from 4.9 to 106.8 months, and the total OT ranged from 106.2 to 258 min in four publications. Five out of eight studies reported LOSs (3.5 to 7.8 days), and there were very limited data on readmissions and reinterventions. There were no IOCs reported, and only one conversion (3.0%) occurred in Meehan et al.’s study [16,20,21,23,25,27,28,29].

### 3.5. Robotic Systems and Port Placements

**Figure 2:**
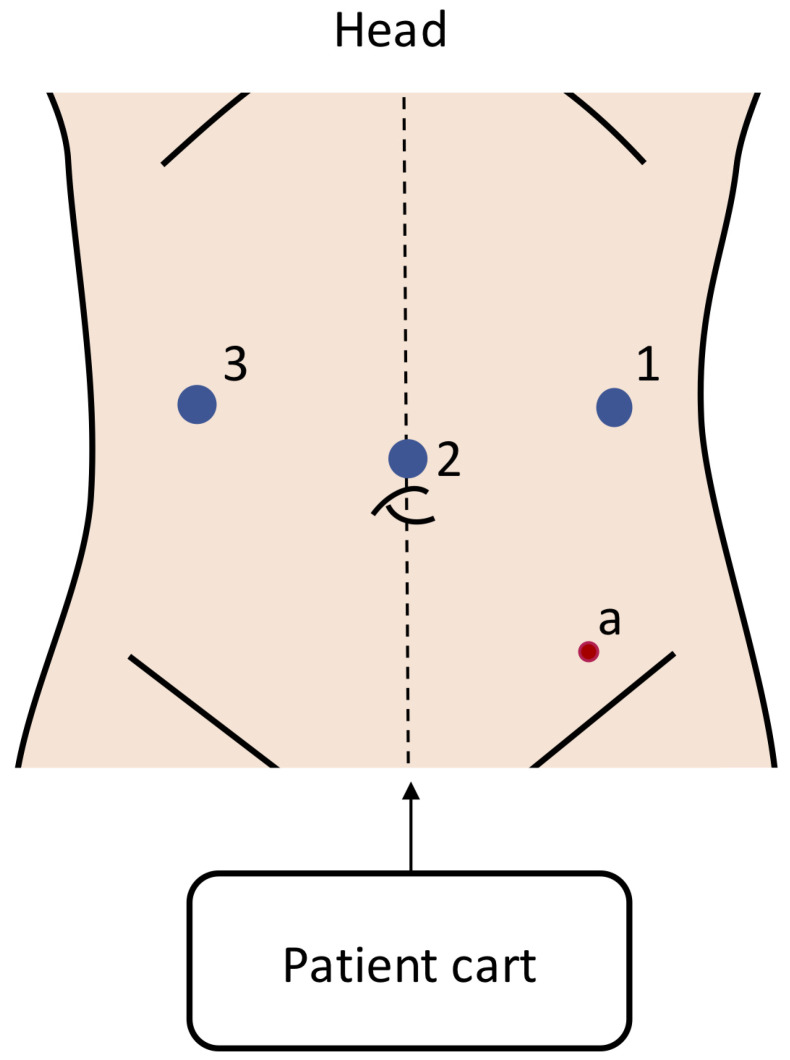
Port placement in robotic surgery for Hirschsprung’s disease. This is the most common setup reported in the included studies [15,17,18,19,22,24,26], with the ports varying between a 5 and 8 mm size depending on the surgical system used. Blue circles: robotic ports; red circle: assistant ports.

All included studies used the Da Vinci Surgical Systems (Intuitive Surgical, Sunnyvale, CA, USA), and were split between the first- and third-generation systems. No other robotic platforms were described in any of the included studies. Six studies used the first-generation Da Vinci platform (five Da Vinci Surgical System and one Da Vinci Standard), and seven studies used the third-generation Da Vinci Si. Two studies did not specify which platform was used. No study reported the use of a second- or fourth-generation Da Vinci platform. Port placements were predominantly a three-arm robotic setup with an assistant port in the lower left abdomen along the midclavicular line. The placements of the robotic ports involved camera ports at the umbilicus (or in case of smaller children, a few cm above it) and the instrument ports at or slightly above the umbilical line. The patient cart was positioned at the patient’s feet. The most used port placement for the surgical treatment of Hirschsprung’s disease is shown in Figure 2.

### 3.6. Methodological Quality

The results of the critical assessment for each study are reported in Table 4 according to the NOS [14]. One study was of high quality (7/9) according to the NOS interpretations, all other studies were of poor quality due to scoring low in “comparability”. The median NOS score for included studies was 4/9 [Q1: 3/9–Q3: 4.75/9]. 

## 4. Discussion

Although the use of robotics in the surgical treatment of paediatric gastrointestinal diseases is on the rise, there are still very few robust data available on the potential benefits. This is common for paediatric congenital diseases and other types, due to their rarity and the limited number of centres capable of adequately performing surgical treatment for these patients. In the present systematic search, fifteen publications were identified with data on the use of robotic surgery for children ≤12 years old, with seven publications on Hirschsprung’s disease. The remaining eight publications showed a multitude of surgical indications, as well as high heterogeneity in population, treatment, and outcomes. 

Only one study reported on comparative data, Li et al., who performed a three-way comparison between laparoscopic, TU-LESS, and robotic surgery for Hirschsprung’s disease. They reported shorter OTs in the laparoscopic and TU-LESS cohorts compared to that of the robotic surgery cohort (152 and 162 vs. 180 min, *p* < 0.001), with no significant differences in blood loss, LOS, IOC, POC, or conversion. The authors also assessed the “Scar Cosmesis Assessment and Rating (SCAR)” [30] for their patients, with statistically significant differences between cohorts. The robotic cohort had the highest overall SCAR score with 4, followed by laparoscopic with 3, and the TU-LESS cohort with 0 (*p* < 0.001). There was no significant difference in faecal incontinence in the long-term follow-up between the cohorts.

Overall, the included studies on Hirschsprung’s disease showed some heterogeneity, with wide ranges in average age at procedure, sex, operative time, and LOS. IOCs, conversion, readmissions, and reinterventions were consistently low across the studies, though this could be influenced by the follow-up time and quality of reporting. The robotic OT was similar compared to that in the laparoscopic and open data reported in a 2015 meta-analysis (Scholfield et al. [31]), though this meta-analysis was assessed with the Duhamel technique rather than the Soave [31]. Their reported OTs were 230 and 245 min for the open and laparoscopic cohorts, with the robotic cohorts in this review ranging from 93.2 to 372 min. Faecal incontinence varied between 0% and 22.2%, with 7/9 studies reporting incontinence less than 7%. The faecal incontinence rate in these robotic cohorts is similar to those published in a recent meta-analysis on the use of laparoscopic surgery for treating Hirschsprung’s disease (Tomuschat et al.) [32]. The low incidence of IOCs, conversion, and comparable long-term outcomes suggest that robotics is a safe approach for paediatric patients, although further high-quality multicentric studies are needed to confirm this hypothesis and identify potential benefits. Interestingly, no comparison with other standardised techniques for Hirschsprung’s disease, such as the transanal endorectal pull-through described by De La Torre and Langer [33,34,35], was reported in the literature. This may be due to the fact that the included studies were performed in centres specialised in MIS. However, comparisons with other techniques would be very relevant and interesting for this topic, in order to identify the strengths and weaknesses in perioperative and long-term outcomes for this niche population.

The remaining publications retrieved were very divided in surgical indication, procedure, and the reporting of outcomes. Two studies, Al-Bassam et al.’s and Chang et al.’s, reported on ARMs, but had a cumulative sample size of just 22 patients with no comparator cohort. This limits the strength of the reported outcomes. Their average age at surgery ranged from 4.9 to 6 months, and only Al-Bassam et al. reported on OT with 214 (130–305) minutes. The authors had no conversions or IOCs, but did not report on POCs, readmissions, or reinterventions, limiting the quality of the reports. 

The remaining studies were split up amongst a multitude of diseases (achalasia, mesenteric cysts, the redo of Hirschsprung’s disease, and restorative proctocolectomy), age ranges (8.0 to 106.8 months), and sex (0% to 100% male). The indications were rare and niche, and the sample sizes so small that comparing them to those in the existing literature for other surgical techniques is of limited to no benefit. There is a real need for the pooling of data from multiple expert centres on the use of robotic surgery for these complex diseases, and to assess potential benefits for these high-risk populations. The establishment of multicentric prospective databases or registries would be beneficial.

All reports in the present systematic review properly reported on IOCs and conversion, with very limited occurrences suggesting the safety and feasibility of the use of robotics in these complex patients. 

All the studies included in the review reported the safe use of first- and third-generation Da Vinci platforms. These platforms are currently considered obsolete compared with the technical advancements from the fourth generation (X and Xi). These multiquadrant four-arm robots could potentially be beneficial in the narrow abdomen of children as they are in the narrow cavities of the pelvis or the neck [36]. However, the role of the fourth arm should be investigated since the space for the port placement could be minimal especially in small children or newborns with high risk of external or internal clashes. The X and Xi platforms use 8 mm ports, which are still wider than their laparoscopic counterpart, and have longer instrument shafts compared to the previous generations, which are not essential in paediatric patients. The role of novel modular platforms such as the Versius Robotic System (CMR Surgical, Cambridge, UK) with its 5 mm ports and miniaturised arms could be beneficial in paediatric patients; however, this should be properly investigated and trialed. The first-generation Hugo Robotic System (Medtronic, Minneapolis, MN, USA), with a three robotic arm setting, could be adopted in paediatric surgery, but it still lacks 5 mm instruments, energy, and staplers. A clear key player in robotic surgery could be the adoption of the Da Vinci Single Port (SP) platform with its 25 mm single-port cannula with four articulated instruments [29]. This would allow the surgeon to comfortably have four articulated instruments under their direct control with the potential to avoid the use of the assistant port. However, the port dimension could be too wide for very young children with potential risk of abdominal wall weakening and possible risk of incisional hernias. On the other hand, Li et al. reported on the use of laparoscopic single-site surgery in their TU-LESS cohort with the use of a glove port and did not observe increased POCs compared to the other cohorts. The Da Vinci SP platform could be beneficial to the application of single-site surgery similar to their TU-LESS cohort and could increase flexibility and manoeuvrability in these small compartments. Finally, the use of advance insufflators such as the Airseal (Conmed, Largo, FL, USA) could be beneficial in the small abdomen allowing a low-pressure surgery with no fumes, potentially reducing the length of surgery and the risk of anaesthesiologic complications. The development of novel miniaturised single-port platforms such as the MIRA (Virtual Incision, Lincoln, NE, USA) and the Vicarious Surgical single-port platform (Waltham, MA, USA), with its 15 mm port and increased freedom of movement, should be strongly kept in consideration for paediatric surgery. 

The strength of this review is found in the systematic approach to identifying relevant publications, its thorough retrieval of all relevant data in the included publications, and the inclusion criteria maintained to promote consistency across the review. This last point is reflected in only including data for children ≤12 years old, to reinforce the fact that the authors aimed to assess the use of robotic surgery in paediatric gastrointestinal diseases only, and to assess challenges in using robotics for this population. Weaknesses in this review include the low quality of data and (retrospective) study designs in the inclusions, the lack of comparator cohorts in most publications, and the heterogeneity across the board, preventing the use of data pooling and meta-analyses. Furthermore, all included studies used first- and third-generation of Da Vinci robotic platforms and did not provide accurate details on instrument usage. This, along with the fact that the latest generation of Da Vinci platforms and other robotic platforms are not reported in the existing literature, is another limitation of this review, and reiterates the need for further studies on these surgical innovations in paediatric surgery.

The critical assessment for the risk of bias was low in all studies except for Li et al.’s [17] due to scoring low in “comparability”, which is a limitation acknowledged by the creators of the NOS. The median NOS score was 4/9, and ranged from 2/9 to 7/9, suggesting that if comparability was not a factor, most included studies would have scored as fair or good quality. Unfortunately, this assumption cannot be supported through external validation, and thus the reviewers have accepted the score as represented by the NOS [14].

## 5. Conclusions

As for the adult population, robotic paediatric surgery is on the rise, and technological advancements and innovations will open further possibilities. However, in contrast to adult patients, robotic surgery is still rarely used in paediatric cases. Therefore, not enough data are available to comment on the potential benefits and risks compared to conventional minimally invasive approaches. Further technological developments and research are needed to enhance our understanding of the potential that robotics may hold for the field of paediatric surgery. 

## Figures and Tables

**Table 1:** Studies on robotic surgery for paediatric gastrointestinal diseases. RSC = retrospective single-centre; PSC = prospective single-centre; PMC = prospective multi-centre; NA = Not Applicable; IQR = Interquartile range; ARM = anorectal malformation [15,16,17,18,19,20,21,22,23,24,25,26,27,28,29].

Author, Year	Country	Study Design	Sample Size, n	Time of Data	Median Age (Range), Months	Disease	Robotic Platform	Generation
Altokhais, 2016 [20]	Saudi Arabia	RSC	6	January 2004–November 2015	84 (24–144)	Achalasia	Da Vinci Si	3rd
Al-Bassam, 2011 [28]	Saudi Arabia	RSC	5	April 2006–March 2010	6 (4–11)	ARM	Da Vinci Surgical System	1st
Chang, 2021 [23]	China	RSC	17	October 2016–January 2018	Median NA (3–9)	ARM	Da Vinci Si	3rd
Li, 2022 [17]	China	PSC	90	2015–2019	4.2 (range NA)	Hirschsprung’s disease	NA	NA
Quynh, 2020 [15]	Vietnam	RSC	55	December 2014–December 2017	24.5 (6–120)	Hirschsprung’s disease	Da Vinci Surgical System	1st
Hebra, 2011 [19]	United States	RSC	12	2003–2009	3.7 (1.4–7.4)	Hirschsprung’s disease	Da Vinci Surgical System	1st
Mattioli, 2017 [18]	Italy	RSC	2	NA	20.0–60.0	Hirschsprung’s disease	Da Vinci Si	3rd
Delgado-Miguel, 2021 [22]	United States	PSC	15	2011–2020	4 [IQR 3–6]	Hirschsprung’s disease	Da Vinci Si	3rd
Zhang, 2021 [24]	China	PMC	156	July 2015–January 2022	9.5 (0.6–132.0)	Hirschsprung’s disease	Da Vinci Si	3rd
Prato, 2019 [26]	Italy	RSC	9	October 2015–June 2019	24 (12–120)	Hirschsprung’s disease	Da Vinci Si	3rd
Chen, 2023 [16]	China	RSC	12	February 2021–August 2022	69.7 (18.2–155.0)	Mesenteric cysts	NA	NA
Meehan, 2009 [29]	United States	RSC	33	October 2002–September 2007	8.0 (0–27.0)	Multiple indications	Da Vinci Standard	1st
Alotaibi, 2018 [27]	Saudi Arabia	RSC	49	June 2004–November 2013	NA	Multiple indications	Da Vinci Surgical System	1st
Prato, 2020 [25]	Italy	RSC	4	January 2012–January 2020	78 (range NA)	Redo Hirschsprung’s disease	Da Vinci Si	3rd
Romeo, 2021 [21]	Italy	PSC	5	2015–2016 and 2019–2021	106.8 (43.2–141.6)	Restorative proctocolectomy	Da Vinci Surgical System	1st

**Table 2:** Hirschsprung’s disease. Data are presented as the absolute value (percentage), mean ± SD, median [Q1–Q3], or median (min–max). For comparative studies, variables with significant differences are indicated with *. OT = operative time; LOS = length of stay (in days); IOC = intra-operative complication; POC = postoperative complication; Rob = robotic; Lap = laparoscopic; TU-LESS = transumbilical laparoscopy single-site surgery; NA = Not Applicable [15,17,18,19,22,24,26].

Author, Year	Surgical Technique	Procedure	Age, Months	Male Sex	OT, Mins	IOC (%)	LOS, Days	POC	Conversion	Re-Admissions	Re-Interventions	Fecal Incontinence	Follow-Up, Months
Li, 2022 [17]	Rob	Soave	4.3 ± 1.4	18 (64.3%)	180 ± 21 *	0 (0%)	8.4 ± 0.6	7 (25.0%)	0 (0%)	1 (3.6%)	1 (3.6%)	2 (7.1%)	NA
	Lap		4.3 ± 1.3	20 (66.7%)	152 ± 21 *	0 (0%)	8.5 ± 0.9	7 (23.3%)	1 (3.3%)	2 (6.7%)	1 (3.3%)	2 (6.7%)	NA
	TU-LESS		4.1 ± 1.5	22 (68.8%)	162 ± 22 *	0 (0%)	8.8 ± 0.9	9 (28.1%)	1 (4.5%)	3 (9.4%)	1 (3.1%)	1 (3.1%)	NA
Quynh, 2020 [15]	Rob	Soave	24.5 (6–120)	44 (80.0%)	93.2 ± 35	0 (0%)	5.5 (4–8)	6 (10.9%)	0 (0%)	NA	NA	2 (3.6%)	43.2 (30–66)
Hebra, 2011 [19]	Rob	Swenson	3.7 (1.4–7.4)	NA	230	1 (8.3%)	3	3 (25.0%)	NA	NA	NA	NA	36
Mattioli, 2017 [18]	Rob	Soave	20.0–60.0	1 (50%)	337.5 ± 152	0 (0%)	6.0 ± 1.4	0 (0%)	0 (0%)	0 (0%)	0 (0%)	0 (0%)	4.5 ± 2.1
Delgado-Miguel, 2021 [22]	Rob	Soave	4 [3–6]	9 (60%)	240 ± 72	0 (0%)	3 [3–4]	3 (20.0%)	0 (0%)	0 (0%)	1 (6.7%)	0 (0%)	79 [45–115]
Zhang, 2021 [24]	Rob	Soave	9.5 (0.6–132.0)	NA	155.2 ± 16.8	0 (0%)	7.3 ± 1.7	25 (16.0%)	0 (0%)	NA	2 (1.3%)	7 (4.5%)	44.0 [6.0–78.0]
Prato, 2019 [26]	Rob	Soave	24 (12–120)	NA	372 ± 76	4 (44.4%)	7 (4–10)	3 (33.3%)	0 (0%)	NA	NA	2 (22.2%)	12 [5–20]

**Table 3:** Other diseases. Data are presented as the absolute value (percentage), mean ± SD, median [Q1–Q3], or median (min–max). LOS = length of stay (in days); IOC = intraoperative complication; POC = postoperative complication; NA = Not Applicable [16,20,21,23,25,27,28,29].

Author, Year	Disease	Age, Months	Male Sex	OT, Mins	IOC	LOS, Days	POC	Conversion	Re-Admissions	Re-Interventions	Follow-Up, Months
Altokhais, 2016 [20]	Achalasia	84 (24–144)	2 (33.3%)	204 (186–250)	0 (0%)	3.5 (2–7)	0 (0%)	0 (0%)	NA	NA	24 (6–132)
Al-Bassam, 2011 [28]	ARM	6 (4–11)	5 (100%)	214 (130–305)	0 (0%)	6 (5–7)	NA	0 (0%)	NA	0 (0%)	12 (6–36)
Chang, 2021 [23]	ARM	4.9	17 (100%)	NA	0 (0%)	10 (7–14)	4 (23.5%)	0 (0%)	NA	NA	12
Chen, 2023 [16]	Mesenteric cysts	69.7 (18.2–155.0)	8 (66.7%)	106.2 ± 33.7	0 (0%)	7.8 ± 3.3	2 (16.7%)	0 (0%)	NA	NA	NA
Meehan, 2009 [29]	Multiple indications	8.0 (0–27.0)	NA	NA	NA	NA	NA	1 (3.0%)	1 (3.0%)	1 (3.0%)	NA
Alotaibi, 2018 [27]	Multiple indications	NA	NA	NA	0 (0%)	NA	NA	NA	NA	NA	NA
Prato, 2020 [25]	Redo Hirschsprung’s disease	78	3 (75%)	NA	0 (0%)	NA	NA	0 (0%)	NA	NA	3.75 (1–16)
Romeo, 2021 [21]	Restorative proctocolectomy	106.8 (43.2–141.6)	0 (0%)	258 ± 54	0 (0%)	7.4 ± 4.4	1 (20%)	0 (0%)	NA	NA	0.6 (0.3–5.9)

**Table 4:** Quality appraisal (risk of bias) for included articles using the Newcastle Ottawa Scale [14]. Good quality: three or four stars in the selection domain AND one or two stars in the comparability domain AND two or three stars in the outcome/exposure domain. Fair quality: two stars in the selection domain AND one or two stars in the comparability domain AND two or three stars in the outcome/exposure domain. Poor quality: zero or one stars in the selection domain OR zero stars in the comparability domain OR zero or one stars in the outcome/exposure domain. On this scale, the cohort selection methods, at the discretion of the clinicians, lead to poor scores limited by the comparability domain.

Study	Selection				Comparability		Outcomes			Total
	Representativeness of the Exposed Cohort	Selection of the Non-Exposed Cohort	Ascertainment of Exposure	Outcome not Present at the Start of the Study			Assessment of Outcome	Follow-Up Length	Adequacy of the Follow-Up of Cohorts	
Zhang, M., 2023 [24]	*		*				*	*	*	5
Quynh, T.A., 2022 [25]	*		*				*	*	*	5
Chang, X., 2022 [23]	*		*					*	*	4
Li, W., 2022 [17]	*	*	*		*		*	*	*	7
Chen, X., 2022 [16]	*		*				*			3
Romeo, C., 2022 [21]	*		*				*			3
Delgado-Miguel, C., 2022 [22]	*		*				*	*	*	5
Pini Prato, A., 2020 (JLAST) [25]	*		*					*		3
Pini Prato, A., 2020 (Ped Surg Int) [26]	*		*					*	*	4
Alotaibi, W., 2019 [27]	*		*				*			3
Mattioli, G., 2017 [18]	*		*							2
Altokhais, T., 2016 [20]	*		*				*	*	*	5
Hebra, A., 2011 [19]	*		*				*	*		4
Albassam, A., 2011 [28]	*		*				*	*		4
Meehan, J., 2009 [29]	*		*							2

## Data Availability

The datasets used or analysed during the current study are available from the corresponding author upon reasonable request. The data are not publicly available due to the fact that no funding was sought for this study and therefore there was no capacity to publish the data online.

## References

[1] Hall N.J., Pacilli M., Eaton S., Reblock K., A Gaines B., Pastor A., Langer J.C., I Koivusalo A., Pakarinen M.P., Stroedter L. (2009). Recovery after open versus laparoscopic pyloromyotomy for pyloric stenosis: A double-blind multicentre randomised controlled trial. Lancet.

[2] Zitsman J.L. (2003). Current concepts in minimal access surgery for children. Pediatrics.

[3] Zitsman J.L. (2006). Pediatric minimal-access surgery: Update 2006. Pediatrics.

[4] Georgeson K.E., Owings E. (2000). Advances in minimally invasive surgery in children. Am. J. Surg..

[5] Mattei P. (2007). Minimally invasive surgery in the diagnosis and treatment of abdominal pain in children. Curr. Opin. Pediatr..

[6] Standard. https://www.davincisurgerycommunity.com/systems_i_a/standard.

[7] Meininger D., Byhahn C., Markus B.H., Heller K., Westphal K. (2001). Roboterassistierte, endoskopische fundoplikatio nach nissen bei kindern: Hämodynamik, gasaustausch und anästhesiologisches management. Anaesthesist.

[8] Cundy T.P., Shetty K., Clark J., Chang T.P., Sriskandarajah K., Gattas N.E., Najmaldin A., Yang G.-Z., Darzi A. (2013). The first decade of robotic surgery in children. J. Pediatr. Surg..

[9] Richards H.W., Kulaylat A.N., Cooper J.N., McLeod D.J., Diefenbach K.A., Michalsky M.P. (2021). Trends in robotic surgery utilization across tertiary children’s hospitals in the United States. Surg. Endosc..

[10] Mahida J.B., Cooper J.N., Herz D., Diefenbach K.A., Deans K.J., Minneci P.C., McLeod D.J. (2015). Utilization and costs associated with robotic surgery in children. J. Surg. Res..

[11] Geller E.J., Matthews C.A. (2013). Impact of robotic operative efficiency on profitability. Am. J. Obstet. Gynecol..

[12] Page M.J., McKenzie J.E., Bossuyt P.M., Boutron I., Hoffmann T.C., Mulrow C.D., Shamseer L., Tetzlaff J.M., Akl E.A., Brennan S.E. (2021). The PRISMA 2020 statement: An updated guideline for reporting systematic reviews. BMJ.

[13] Ouzzani M., Hammady H., Fedorowicz Z., Elmagarmid A. (2016). Rayyan—A web and mobile app for systematic reviews. Syst. Rev..

[14] Wells G.A., Shea B., O’Connell D., Peterson J., Welch V., Losos M., Tugwell P. Ottawa Hospital Research Institute. https://www.ohri.ca/programs/clinical_epidemiology/oxford.asp.

[15] Quynh T.A., Hien P.D., Du L.Q., Long L.H., Tran N.T.N., Hung T. (2022). The follow-up of the robotic-assisted Soave procedure for Hirschsprung’s disease in children. J. Robot. Surg..

[16] Chen Q., Zhang S., Luo W., Cai D., Zhang Y., Huang Z., Xuan X., Xiong Q., Gao Z. (2023). Robotic-assisted laparoscopic management of mesenteric cysts in children. Front. Pediatr..

[17] Li W., Lin M., Hu H., Sun Q., Su C., Wang C., Li Y., Li Y., Chen J., Luo Y. (2022). Surgical Management of Hirschsprung’s Disease: A Comparative Study Between Conventional Laparoscopic Surgery, Transumbilical Single-Site Laparoscopic Surgery, and Robotic Surgery. Front. Surg..

[18] Mattioli G., Pio L., Leonelli L., Razore B., Disma N., Montobbio G., Jasonni V., Petralia P., Prato A.P. (2017). A provisional experience with robot-assisted Soave procedure for older children with hirschsprung disease: Back to the future?. J. Laparoendosc. Adv. Surg. Tech..

[19] Hebra A., Smith V.A., Lesher A.P. (2011). Robotic Swenson pull-through for Hirschsprung’s disease in infants. Am. Surg..

[20] Altokhais T., Mandora H., Al-Qahtani A., Al-Bassam A. (2016). Robot-assisted heller’s myotomy for achalasia in children. Comput. Assist. Surg..

[21] Romeo C., Di Fabrizio D., Impellizzeri P., Arena S., Dipasquale V., Palo F., Costa S., Pellegrino S., Antonuccio P., Romano C. (2022). Laparoscopic robotic-assisted restorative proctocolectomy and ileal J-pouch-anorectal anastomosis in children. Pediatr. Surg. Int..

[22] Delgado-Miguel C., Camps J.I. (2022). Robotic Soave pull-through procedure for Hirschsprung’s disease in children under 12-months: Long-term outcomes. Pediatr. Surg. Int..

[23] Chang X., Cao G., Pu J., Li S., Zhang X., Tang S.-T. (2022). Robot-assisted anorectal pull-through for anorectal malformations with rectourethral and rectovesical fistula: Feasibility and short-term outcome. Surg. Endosc..

[24] Zhang M.-X., Zhang X., Chang X.-P., Zeng J.-X., Bian H.-Q., Cao G.-Q., Li S., Chi S.-Q., Zhou Y., Rong L.-Y. (2023). Robotic-assisted proctosigmoidectomy for Hirschsprung’s disease: A multicenter prospective study. World J. Gastroenterol..

[25] Prato A.P., Arnoldi R., Faticato M.G., Mariani N., Dusio M.P., Felici E., Tentori A., Nozza P. (2020). Minimally Invasive Redo Pull-Throughs in Hirschsprung Disease. J. Laparoendosc. Adv. Surg. Tech..

[26] Prato A.P., Arnoldi R., Dusio M.P., Cimorelli A., Barbetta V., Felici E., Barbieri P., Barbero S., Carlini C., Petralia P. (2020). Totally robotic soave pull-through procedure for Hirschsprung’s disease: Lessons learned from 11 consecutive pediatric patients. Pediatr. Surg. Int..

[27] Alotaibi W.M. (2019). Anesthesia experience of pediatric robotic surgery in a University Hospital. J. Robot. Surg..

[28] Albassam A., Gado A., Mallick M.S., Alnaami M., Al-Shenawy W. (2011). Robotic-assisted anorectal pull-through for anorectal malformations. J. Pediatr. Surg..

[29] Meehan J.J. (2009). Robotic surgery in small children: Is there room for this?. J. Laparoendosc. Adv. Surg. Tech..

[30] Kantor J. (2017). Reliability and Photographic Equivalency of the Scar Cosmesis Assessment and Rating (SCAR) Scale, an Outcome Measure for Postoperative Scars. JAMA Dermatol..

[31] Scholfield D.W., Ram A.D. (2016). Laparoscopic Duhamel Procedure for Hirschsprung’s Disease: Systematic Review and Meta-analysis. J. Laparoendosc. Adv. Surg. Tech..

[32] Tomuschat C., Zimmer J., Puri P. (2016). Laparoscopic-assisted pull-through operation for Hirschsprung’s disease: A systematic review and meta-analysis. Pediatr. Surg. Int..

[33] De La Torre-Mondragón L., Ortega-Salgado J.A. (1998). Transanal endorectal pull-through for Hirschsprung’s disease. J. Pediatr. Surg..

[34] Langer J.C., Minkes R.K., Mazziotti M.V., Skinner M.A., Winthrop A.L. (1999). Transanal one-stage soave procedure for infants with Hirschsprung’s disease. J. Pediatr. Surg..

[35] De La Torre L., Langer J.C. (2010). Transanal endorectal pull-through for Hirschsprung disease: Technique, controversies, pearls, pitfalls, and an organized approach to the management of postoperative obstructive symptoms. Semin. Pediatr. Surg..

[36] Piozzi G.N., Baek S.J., Kwak J.M., Kim J., Kim S.H. (2021). Anus-Preserving Surgery in Advanced Low-Lying Rectal Cancer: A Perspective on Oncological Safety of Intersphincteric Resection. Cancers.

